# Feats of supercontractile strength: functional convergence of supercontracting muscle properties among hyoid musculature in chameleons

**DOI:** 10.1098/rspb.2025.0078

**Published:** 2025-03-26

**Authors:** Nikole G. Schneider, Nicholas A. Henchal, Raul E. Diaz Jr, Christopher V. Anderson

**Affiliations:** ^1^ Department of Biology, University of South Dakota, Vermillion, SD 57069, USA; ^2^ Department of Biological Sciences, California State University Los Angeles, Los Angeles, CA 90032, USA

**Keywords:** skeletal muscle architecture, Chamaeleonidae, superelongation, contractile physiology, sarcomere length inhomogeneity

## Abstract

The structure of sarcomeres imposes limits to the capacity of striated muscle to change length and produce force, with z-disc and myosin filament interactions constraining shortening. Conversely, supercontracting muscles, hitherto only known among vertebrates in the tongue retractor muscle (m. hyoglossus) of chameleons, have perforated z-discs that allow myosin filaments to extend through them into adjacent sarcomeres, permitting continued shortening and force development. Additional hyolingual muscles in chameleons undergo extreme length changes during feeding as well and may benefit from supercontractile properties. We compared length–tension relationship data and transmission electron microscopy images from four chameleon muscles to test for the presence of additional supercontracting muscle. We document the second known example of a supercontracting muscle among vertebrates (the m. sternohyoideus superficialis) and show that the m. sternohyoideus profundus exhibits functional convergence with supercontracting muscles by increasing the range of muscle lengths over which it can exert force through the exploitation of sarcomere length non-uniformity across its muscle fibres. Additionally, we show that chameleon supercontracting muscles may share common contractile and structural properties due to a common origin from occipital somites. These results provide important insights into the developmental and evolutionary patterns associated with supercontracting muscle and extreme muscle elongation.

## 1. Introduction

Sarcomeres are the structural and functional units of skeletal muscle in animals. When activated, sarcomeres generate the force, or tension, underlying muscle contraction [1,2]. Force development causes the muscles to contract isometrically (no length change), concentrically (shortening) or eccentrically (lengthening) depending on external forces acting on the muscle. This tension is generated by the interaction of actin thin filaments and myosin thick filaments via crossbridges, with additional contributions by the protein titin and other sarcomere elements. These forces can cause the filaments to slide along each other’s lengths, resulting in length change and ultimately powering movement [1–6]. The contractile force of striated muscle is maximized across a narrow range of intermediate lengths, where actin and myosin filament overlap allows for all available myosin crossbridges to bind to actin binding sites [7,8]. When a sarcomere is lengthened beyond this intermediate length, the overlap between the myosin and actin filament binding sites gradually declines, resulting in their ability to exert force diminishing until the filaments no longer overlap and no force can be produced [7,8]. Conversely, as sarcomeres shorten beyond these intermediate lengths and filaments from opposite sides of the sarcomere interact, force production is rapidly diminished. This decrease in force development is traditionally thought to be caused by the physical compression of myosin filaments with the rigid z-discs at either end, which support the sarcomere structure [7–9]. However, due to the discovery of the mesh-like structure of z-discs, new hypotheses propose that filaments may extend into the z-discs at short lengths, with force declining due to possible inter-sarcomere interactions of titin and other filaments [10–12]. The extent to which striated muscle is able to change length and still produce force is thus limited by this length–tension relationship, with typical vertebrate skeletal muscle limited to reversibly shortening only up to 50% of its resting length [7,9,13], although some evidence of force production at shorter lengths, possibly due to myosin sliding through the z-discs, has been shown from electron micrographs of muscle following contractions at short lengths [12].

As sarcomeres are arranged in series down the length of muscle fibrils, their individual length changes are additive to achieve larger muscle excursion distances [14]. Larger absolute muscle excursions in vertebrates, therefore, tend to be associated with longer muscle fibres, and thus, more sarcomeres in series. Cases of extreme length change, however, would be difficult to achieve when the space to store muscles long enough to power these length changes is limited. Indeed, some plethodontid salamanders are able to ballistically project their tongue up to 80% of their body length [15] and then retract the tongue and prey back into the mouth. This projection is accomplished by their tongue retractor muscle originating on the pelvis, dramatically increasing fibre lengths over analogous tongue muscles in other tetrapods that originate on the hyoid [15–17].

In contrast to increasing the number of sarcomeres in series, some muscles have alternate mechanisms that allow filaments to continue to interact with each other over greater lengths. For instance, the muscle fibres of pennate muscles may undergo rotation during contraction, thereby changing the degree to which sarcomere length change corresponds with whole-muscle length changes [18]. Further, while vertebrate muscle sarcomere lengths are quite uniform (typically 2.0−2.8 µm), invertebrate skeletal muscle sarcomere lengths are highly variable (1.9−17.8 µm in arthropods and up to 40 µm in annelids), with longer sarcomeres also having longer myosin and actin filaments [19]. These longer filament lengths allow for higher force production due to a greater number of crossbridges, and increased length changes as sarcomeres can lengthen further before filaments no longer overlap. Smooth muscle, on the other hand, does not contain discrete sarcomeres or z-discs. The lack of these features allows for greater length change in the muscle; however, the qualities of smooth muscle limit its velocity and force output when shortening [20]. Conversely, obliquely striated muscle in invertebrates such as cephalopods [21–23], nematodes [24,25] and annelids [26,27] have discrete sarcomeres with z-discs (dense bodies) arranged at oblique angles, allowing for shearing of the myosin filaments to increase overlap when contraction occurs [24]. In some cases, these obliquely striated fibres allow for muscles to operate at long lengths beyond those that would normally be possible by typical skeletal muscle, occasionally referred to as superelongation [28]. Finally, supercontracting muscles, which are typically defined as striated muscle able to naturally contract below 50% of resting length [29,30] have perforations in their z-discs, allowing the myosin filaments to slide past the z-disc borders into adjacent sarcomeres and continue to interact with actin filaments [9,31]. The ability to shorten beyond typical sarcomere lengths allows for extensive overlap of actin and myosin filaments at maximum muscle extension, retaining high force production at long muscle lengths [31]. Further, this seems to allow supercontracting muscle to rest at short lengths while maintaining high force capacity, extending the range of lengths over which the muscle is able to lengthen and maintain force due to sustained filament overlap [31,32]. Although supercontracting muscle is commonly found among invertebrates, most notably in the barnacle [29,33,34] and a variety of insects [35–37], the only known supercontracting muscle among vertebrates is the tongue retractor muscle, m. hyoglossus, in chameleons [9,31,32]. Similar to some plethodontid salamanders, chameleons use a ballistic tongue projection mechanism to capture prey from distances of up to two and a half body lengths away [38]. Their tongue retractor muscle, however, originates on the hyoid, which, due to its cranial location, necessitates the muscle to operate beyond the intermediate length changes permitted under typical vertebrate sarcomere structure [17,39,40].

**Figure 1.**
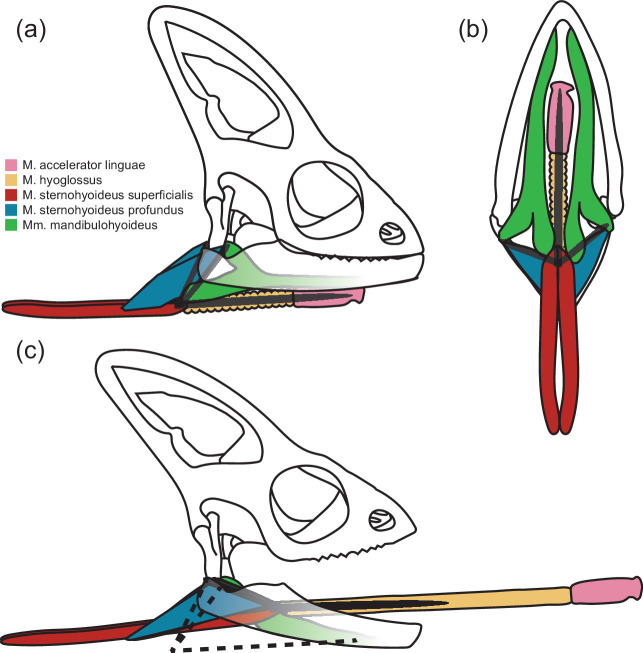
Anatomical illustrations showing the location of important hyoid and tongue muscles in chameleons. Muscles are displayed at rest (*a, b*) and during tongue projection (*c*) in lateral (*a, c*) and ventral (*b*) views. The skull of a chameleon (white) is depicted with colour-coded muscles overlaid. The hyoid apparatus is illustrated as dark lines with two ceratobranchials posterolaterally and a single midline entoglossal process ventromedially. The hyoid apparatus is illustrated in its protruded configuration in (*c*) with dotted lines approximating the resting configuration.

Whereas the m. hyoglossus in chameleons, which originates on the medial side of the ceratobranchials and inserts on the lateral aspects of the connective tissue surrounding the m. accelerator linguae of the tongue [39,41], is known to undergo considerable length changes during tongue projection and retraction, other muscles associated with the hyoid apparatus also undergo impressive excursions (figure 1). In particular, prey transport in chameleons exhibits an increased dependence on the hyoid, with more extensive movements than other lizards [17]. It has been noted that to produce these movements, the hyoid retractors (mm. sternohyoideus) must be contracting more than 50% of their resting length and thus may be supercontracting as well [17]. The m. sternohyoideus superficialis originates on the ventral side of the xiphisternum and inserts on the ventral side of the basihyal with no obvious tendon or aponeurosis at either attachment, whereas the m. sternohyoideus profundus, *sensu* Meyers *et al*. [42], originates on a connective tissue band directly anterior to the xiphisternum and inserts on the posterior distal half of the ceratobranchials with no obvious tendon or aponeurosis [39,41]. Both divisions of the mm. sternohyoideus retract the hyoid apparatus, however the m. sternohyoideus profundus is also involved in unfolding the hyoid during tongue protrusion, whereby the angle between the ceratobranchial and entoglossal process increases from an acute to obtuse angle [39,40,43,44].

We predicted that because chameleons already possess the capacity for supercontracting muscle (i.e. the m. hyoglossus), this pathway could be co-opted [45] by other muscles to express supercontractile properties as well. Based on their large excursions, we hypothesized that both the mm. sternohyoideus superficialis et profundus would exhibit broadened length–tension relationships compared with those of normal sarcomeres and that both muscles would have perforated z-discs signifying that they too are supercontracting muscles. To test these hypotheses, we collected length–tension relationship data to compare the contractile properties of the mm. sternohyoideus superficialis et profundus in chameleons to their m. hyoglossus, a known supercontracting muscle, and m. triceps scapularis, a representative normal striated muscle originating on the posterior scapula and inserting on the olecranon of the ulna via the scapulohumeral ligament [46]. We then performed transmission electron microscopy (TEM) imaging of these four muscles to analyse muscle ultrastructure and determine the presence or absence of perforated z-discs, a feature that is indicative of supercontracting muscle. Finally, we examined hyoid musculature from a developmental series of chameleon embryos to assess whether observed patterns of supercontracting muscle presence likely represented the development of muscle bellies from common muscle anlages or independent developmental origins.

## 2. Material and methods

### (a) Muscle preparation and contractile physiology

Adult male veiled chameleons (*Chamaeleo calyptratus*) were used to conduct *in vitro* experiments to determine the contractile properties of four muscles: m. triceps scapularis (negative control for supercontracting muscle), m. hyoglossus (positive control for supercontracting muscle), m. sternohyoideus superficialis and m. sternohyoideus profundus. Fifteen specimens were acquired from retail distributors and housed separately in glass terrariums equipped with live plants and a misting system that provided water two to three times daily. Ambient temperature (20–24°C) with incandescent basking lights (30–35°C) and fluorescent ultraviolet (UVB) lighting was provided for 12 h per day. Chameleons were fed a diet of gut loaded crickets three times per week. Husbandry and all experimental procedures associated with muscle contractile physiology were approved by the Institutional Animal Care and Use Committee (IACUC) of the University of South Dakota to C.V.A.

Prior to experimentation, chameleons were euthanized by isoflurane overdose followed by decapitation and double pithing. The muscle of interest was then excised under a dissecting microscope. For the m. triceps scapularis and mm. sternohyoideus superficialis et profundus, the skeletal attachment points were retained with the muscle (electronic supplementary material, figure S1). Silk suture was used to secure the distal attachment of the muscle to the silver chain that connected the muscle to a dual mode servomotor (Model 305C, Aurora Scientific Inc., Ontario, Canada). The opposite bony attachment was secured to the base of the experimental test apparatus (Model 805A, Aurora Scientific Inc., Ontario, Canada) via clamping of the bone (m. triceps scapularis and m. sternohyoideus profundus) or securing the attachment site to the test apparatus with silk suture. Due to the length of the m. hyoglossus and its muscular insertion site, a segment of this muscle was tied off and trimmed away from the full muscle’s length. One end was attached via a silver chain to the servomotor, while the other was secured to the base of the test apparatus (electronic supplementary material, figure S1). During all experiments, muscles were submerged in reptilian Ringer’s solution [47] to mimic body conditions, which was saturated with 100% oxygen and circulated through a temperature-controlled water bath set to the average published selected body temperature of *C. calyptratus* (31–32°C) [48,49]. Muscles were allowed 30 min to acclimate prior to contractile experiments.

Muscles were stimulated via field stimulation using two platinum electrodes attached to the experimental test apparatus on either side of the mounted muscle of interest and connected to a stimulator (Grass S48 stimulator, Grass Medical Instruments, Quincy, MA, USA) and amplifier (Crown DC-300A II amplifier, Crown International Inc., Elkhard, ID, USA). Force and length were sampled from the servomotor at 10 kHz (USB-6361 BNC, National Instruments, Austin, TX, USA; Igor Pro 7, Wavemetrics Inc., Lake Oswego, OR, USA). Each muscle was initially set to a short length with minimal stretch from its unloaded length and thus low passive tension exerted on it. Muscles were then supramaximally stimulated at increasing lengths to create tetanic length–tension curves. Supramaximal stimulation was achieved with 0.2 ms stimulation pulse durations, with stimulation voltage (1.2–3.0 V), frequency (50– 150 pulses per section (pps)) and train duration (150–250 ms) selected individually for each muscle prior to the onset of tetanic muscle contractile experiments. This was achieved first by performing twitch contractions with a stimulation voltage of 0.5 V and incrementally increasing the stimulation voltage by approximately 0.25 V until approximately 50% higher than the voltage force stopped increasing. Tetanic contractions were then performed at 50 pps for 150 ms and those settings adjusted until force traces were smooth and without scalloping and reached a tetanic peak. The length between contractions was then increased by approximately 0.5 mm (0.3 V adjustment on servomotor offset) to approximately 0.9 mm (0.5 V adjustment) and at least seven tetanic contractions were obtained from each muscle (the average number of contractions per muscle ranged from 11 to 15 contractions across the four muscles).

Passive and active forces were quantified, where passive force was measured as the force prior to muscle stimulation onset, and active force was measured as the difference between the passive force and the maximal force exerted during stimulation. Instances where muscles appeared to experience active force declines between stimulations regardless of muscle length were investigated by performing sequential contractions without length change for evidence of fatigue, whereby muscle force production declines considerably across these stimulations. Muscles with fatigue of over 10% active force were determined to have experienced rapid fatigue and excluded from analysis. At the end of each muscle contractile experiment, the muscle length at the optimal length for tetanic contraction (*L*_0_), defined as the length corresponding to the maximal active force produced (peak isometric force; *P*_0_), was measured.

While the calculation of active force by subtracting passive force from the maximal force exerted during stimulation fails to account for potential shifts in *L*_0_ and the potential to underestimate *P*_0_ in muscles with tendons or aponeuroses [50,51], appropriate data on such series elastic components in these muscles are not available to correct for any potential shifts. Further, in our preparations only the m. triceps scapularis exhibits notable series elastic components in their attachment sites, limiting any likely impact of such error on this muscle. While this may result in slightly lower raw *P*_0_ values, shorter calculated *L*_0_ lengths and a slightly narrowed length–tension relationship on the descending limb of the active force curve, it would not be expected to be sufficient to obfuscate differentiation between normalized length–tension relationships of typical striated muscle and the broadened normalized length–tension relationships of supercontracting muscle, particularly on the ascending limb.

### (b) Curve fitting and statistical analysis

**Figure 2.**
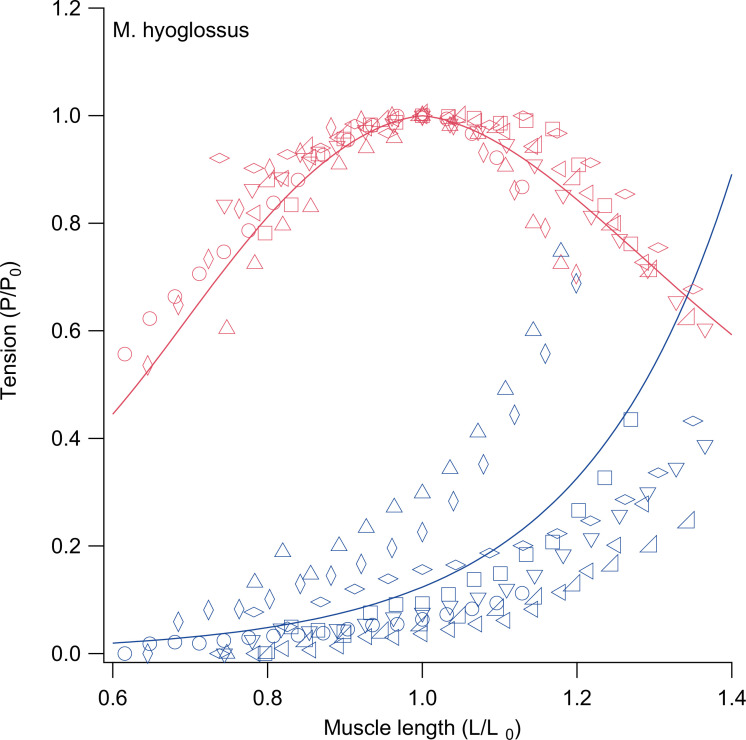
Example of raw active (red) and passive (blue) length–tension data for m. hyoglossus preparations with average fitted curves overlaid. Each individual for which contractile data from this muscle were obtained is represented by a unique shape with muscle average curves calculated as the average of fitted curves for each individual.

After collecting passive and active tension data across a range of muscle lengths, curves were fitted for each individual and an average curve across all individuals was calculated for each muscle of interest (e.g. figure 2). Data were normalized by dividing force values by the peak isometric force for that muscle across muscle lengths (*P*_0_) and dividing length values by the optimal length for tetanic contractions, or the length at the maximum active force (*L*_0_). Passive and active length–tension curves were then fitted following the methods in Anderson & Roberts [52], derived from Otten [53].

From the fitted curves for each individual muscle, the length at which passive tension reached 20% of *P*_0_ (*L*_20_) and the length breadth over which the active tension remained above 80% of *P*_0_ (*F*_80_) were calculated. General linear mixed models with individual as a random effect were used to test for an effect of muscle on *L*_20_ and *F*_80_ values using the nlme package [54], and pairwise post hoc tests for each model were performed in the emmeans package (v. 1.8.9) [55]. All statistical analysis was performed in R Studio (v. 2023.09.1+494) [56] running R v. 4.3.1.

### (c) Histology and muscle ultrastructure

TEM imaging was performed in the Electron Microscopy Core Facility (EMCF) at the University of Nebraska Medical Center. Muscle samples were fixed by immersion in a solution of 2% glutaraldehyde, 2% paraformaldehyde in a 0.1 M sodium cacodylate buffer (pH 7.2) for a minimum of 24 h at 4°C. Samples were then washed with cacodylate buffer three times to rinse away excess fixative. Before processing, samples were post-fixed in a 1% aqueous solution of osmium tetroxide for 1 h and washed again with three buffer exchanges. Subsequently, samples were dehydrated in a graded ethanol series (50/70/90/95/100%) and propylene oxide was used as a transition solvent between the ethanol and embed 812 resin. Samples were then left to sit overnight in a 50:50 propylene oxide:resin solution until all the propylene oxide had evaporated, and incubated in fresh resin for a minimum of 2 h at ambient temperature before final embedding. Polymerization took place at 65°C for 24 h. Thin sections (100 nm) made with a Leica UC7 Ultracut ultramicrotome were placed on 200 mesh copper grids, post-stained with 2% uranyl acetate followed by Reynolds lead citrate, and examined on a Tecnai G2 Spirit TWIN (FEI) operating at an accelerating voltage of 80 kV. Images were acquired digitally with an digital imaging system (AMT Imaging).

Confirmation of supercontracting muscle ultrastructure was determined by the presence of perforated z-discs in the digital imaging of muscle sarcomeres, which appear as discontinuous or dashed bands between adjacent sarcomere units. In contrast, normal striated muscle z-discs present as dark, solid bands separating adjacent sarcomeres.

### (d) Immunohistochemistry

Adult veiled chameleons were previously housed and bred, and embryos collected based on previously described protocols [57,58]. Husbandry, breeding and embryo collection were performed under approved IACUC protocols from La Sierra University to R.E.D.

Embryos were processed for wholemount immunohistochemistry using a horseradish-peroxidase (HRP) secondary as described in Diaz *et al.* [58,59] for MF20 (anti-striated muscle myosin heavy chain1; DSHB no. MF20) and Tuj1 (anti-tubulin beta 3; Covance no. MMS-435P). HRP conjugated secondary immunoglobulin G (IgG) antibodies (goat-anti-mouse IgG [H+L]; Thermo Fisher Scientific no. 31430) were used in conjunction with the DAB (3,3’-diaminodbenzidine) Metal Enhanced Substrate Kit (Thermo Fisher Scientific no. 34065). The successful labelling of later stage chameleon embryos for skeletal muscle (MF20) required the removal of the overlying epidermis of the head and neck using fine forceps (Fine Science Tools; Dumont no. 55) after the first 2 h wash in TN-block solution (1% Bovine Serum Albumen [Omnipure/Calbiochem, 2930] + 0.1M Tris, pH 7.5 + 0.15M NaCl in water) prior to introducing the primary antibody [58,59]. All steps were carried out at 4°C.

## 3. Results

### (a) Muscle contractile physiology

Length–tension curve data from eight m. triceps scapularis, eight m. hyoglossus, five m. sternohyoideus superficialis and four m. sternohyoideus profundus muscle preparations were collected from a total of 15 individuals. From these 15 individuals, successful preparations from two different muscles were collected from 10 individuals. For the remaining five individuals, one muscle preparation from each experienced rapid fatigue during data collection or damage to the muscle during dissection and mounting, resulting in incomplete data collection from that muscle, which was then excluded from analyses. An additional individual had one muscle (the m. sternohyoideus superficialis preparation) removed from subsequent analyses as a statistical outlier using the interquartile range criterion, resulting in four preparations of this muscle included in analyses.

**Figure 3.**
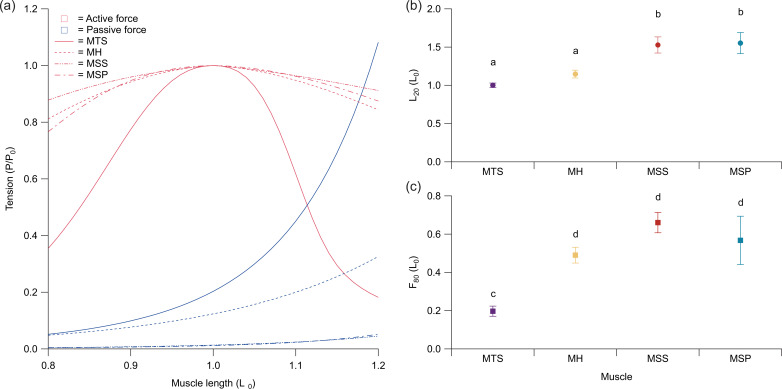
Contractile property comparisons for four chameleon muscles. (a) Average muscle active (red) and passive (blue) length–tension curves calculated for all four muscles. From these curves, comparisons of (b) the relative muscle length at which passive tension reached 20% of peak tetanic tension (*L*_20_) and (c) the relative muscle length breadth at which active tension remained above 80% of peak tetanic tension (*F*_80_) are presented with standard error of the mean (SEM) error bars. Letters (*a-d*) in (b) and (c) represent statistical differences among muscles within each panel. MTS, m. triceps scapularis (purple); MH, m. hyoglossus (yellow); MSS, m. sternohyoideus superficialis (red); MSP, m. sternohyoideus profundus (blue).

The m. hyoglossus, m. sternohyoideus superficialis and m. sternohyoideus profundus exhibited broader average length–tension curves as compared with the m. triceps scapularis (figure 3a; table 1; denoted as MH, MSS, MSP and MTS, respectively). This was demonstrated by the lack of statistical difference between the m. hyoglossus, m. sternohyoideus superficialis and m. sternohyoideus profundus *F*₈₀ values (the length breadth over which the active tension remained above 80% of the muscle’s peak isometric force (*P*_0_)), which each differed significantly from the m. triceps scapularis (figure 3c; tables 1 and 2). The length at which passive tension reached 20% of *P*_0_ (*L*₂₀) exhibited significant similarity between the m. hyoglossus and m. triceps scapularis, and between the m. sternohyoideus superficialis and m. sternohyoideus profundus (figure 3b; tables 1 and 2), with the former two muscles exhibiting stiffer passive tension curves (figure 3a).

**Table 1.** Summary data for muscle dimensions and performance metrics. Values represent average values for each muscle ± standard error around the mean (SEM). MTS, m. triceps scapularis; MH, m. hyoglossus; MSS, m. sternohyoideus superficialis; MSP, m. sternohyoideus profundus.

	MTS	MH	MSS	MSP
muscle mass (g)	0.23 ± 0.03	0.04 ± 0.01	0.17 ± 0.06	0.10 ± 0.04
*P*_0_ (N)	2.18 ± 0.25	0.14 ± 0.02	0.73 ± 0.22	0.43 ± 0.07
*L*_0_ (mm)	28.97 ± 0.97	22.05 ± 1.14	34.51 ± 4.34	26.10 ± 4.10
*L*_20_	1.00 ± 0.03	1.15 ± 0.05	1.53 ± 0.11	1.55 ± 0.14
ascending 80% *P*_0_	0.90 ± 0.01	0.76 ± 0.01	0.74 ± 0.01	0.76 ± 0.06
descending 80% *P*_0_	1.09 ± 0.02	1.28 ± 0.03	1.40 ± 0.05	1.32 ± 0.07
*F*_80_	0.20 ± 0.03	0.49 ± 0.04	0.66 ± 0.05	0.57 ± 0.13

*L*_0_, muscle length at peak isometric force (*P*_0_); *L*_20_, normalized muscle length at which passive tension reached 20% of *P*_0_; ascending 80% *P*_0_, normalized muscle length at which the ascending limb of the active tension trace reached 80% of *P*_0_; descending 80% *P*_0_, normalized muscle length at which the descending limb of the active tension trace reached 80% of *P*_0_; *F*_80_, range of normalized muscle lengths between which active force remained above 80% of *P*_0_.

**Table 2.** Results from Tukey’s HSD (honestly significant difference) analyses examining differences in length–tension curve characteristics between muscles. MSS, m. sternohyoideus superficialis; MSP, m. sternohyoideus profundus; MH, m. hyoglossus; MTS, m. triceps scapularis. Bold adjusted *p-*values indicate the significant difference between muscles.

muscle comparison	*L*_20_ adjusted *p*‐value	*F*_80_ adjusted *p*‐value
MH–MSS	**0.0321**	0.2633
MH–MSP	**0.0257**	0.7853
MH–MTS	0.4055	**0.0187**
MTS–MSS	**0.0082**	**0.0055**
MTS–MSP	**0.0065**	**0.0162**
MSS–MSP	0.9963	0.7679

*L*_20_, normalized muscle length at which passive tension reached 20% of *P*_0_; *F*_80_, range of normalized muscle lengths between which active force remained above 80% of *P*_0_; *P*_0_, peak isometric force.

### (b) Histology/muscle ultrastructure

**Figure 4.**
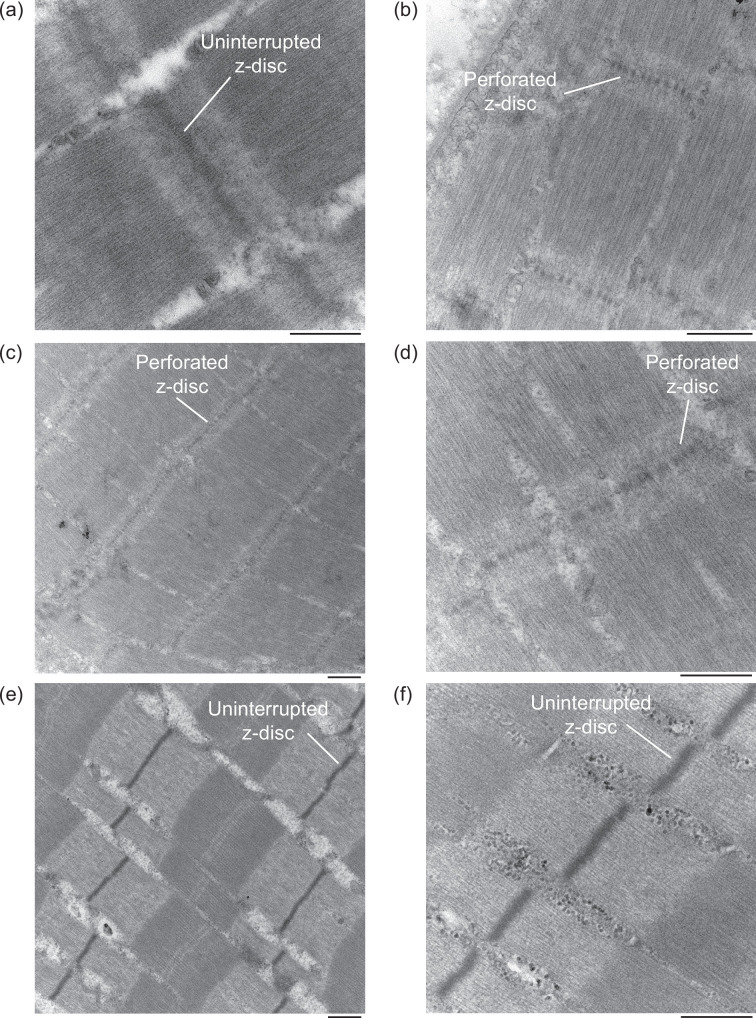
Transmission electron micrographs of chameleon hyoid and tongue muscles. The negative (a; m. triceps scapularis) and positive (b; m. hyoglossus) control for supercontracting muscle exhibit solid and perforated z-discs between adjacent sarcomeres, respectively. The m. sternohyoideus superficialis (*c, d*) exhibits perforated z-discs typical of supercontracting muscles, while the m. sternohyoideus profundus (*e, f*) exhibits solid, uninterrupted z-discs similar to those of typical striated muscle. The scale bar under each image represents 500 nm.

TEM imaging of the negative (m. triceps scapularis) and positive (m. hyoglossus) control for supercontracting muscle revealed solid and perforated z-discs between adjacent sarcomeres, respectively, as would be expected from each muscle (figure 4a,b). The m. sternohyoideus superficialis exhibited perforated z-discs typical of supercontracting muscles and similar to those of the m. hyoglossus (figure 4c,d). Conversely, the m. sternohyoideus profundus exhibited solid, uninterrupted z-discs similar to those of typical striated muscle and those of the m. triceps scapularis (figure 4e,f).

### (c) MF20 antibody staining of embryonic muscle

**Figure 5.**
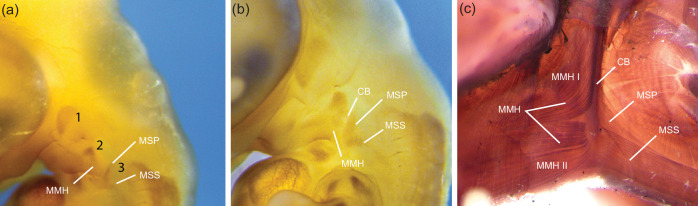
Development of hyoid musculature in MF20 stained chameleon embryos. By 118 days post oviposition (dpo; a,b), hyoid musculature (MF20 positive cells) is visible within the pharyngeal arches, the putative hypoglossal cord region (MMH) and posterior to the ceratobranchials (MSP, MSS). Hyoid musculature in 130 dpo embryos (c) is well defined with adult-like morphology. The MSP remains medial to the MSS at all stages. All embryos are shown in left lateral view. CB, ceratobranchial; MMH, mm. mandibulohyoideus; MMH I, m. mandibulohyoideus I; MMH II, m. mandibulohyoideus II; MSS, m. sternohyoideus superficialis; MSP, m. sternohyoideus profundus.

Hyoid musculature was visualized using the antibody MF20 (Myosin Heavy Chain; DSHB) with the aim of identifying whether the mm. sternohyoideus superficialis et profundus are derived from the same muscle anlage or were of separate origin (figure 5). We examined embryonic veiled chameleons (*Chamaeleo calyptratus*) between 118 and 130 days post oviposition (dpo). MF20 positive cells are seen in pharyngeal arches (PA) I–III/IV as described previously [59]. At 118 dpo, precursor cells for the m. mandibulohyoideus I and II (MMH) arise from a population of MF20 positive cells in the posteroventral (distal) portion of PA II whereas the mm. sternohyoideus profundus et superficialis arise at approximately 90° to each other with the m. sternohyoideus profundus present deeper and in a vertical orientation, appearing to form from distinct precursor MF20 populations in PA III. Intermediate between MMH and the m. sternohyoideus anlage is a vertical MF20 negative bar denoting mesenchyme associated with the differentiating hyoid complex (ceratobranchials). Muscles are clearly defined by 130 dpo, further supporting the deeper presence and distinct muscle anlage origin for the mm. sternohyoideus superficialis et profundus (figure 5).

## 4. Discussion

With typical skeletal muscle sarcomere structure imposing intrinsic limitations on the lengths over which force can be produced, instances of extreme length change necessitate muscle to undergo structural adaptations to power these movements. During ballistic tongue projection in chameleons, the tongue retractor muscle (m. hyoglossus) is able to retract the tongue and prey from distances of up to 2.5 body lengths due to its modified sarcomere structure with perforated z-discs, resulting in what is known as supercontracting muscle [9,17,31,32]. These perforations allow myosin filaments to extend into adjacent sarcomeres and interact with actin filaments at short muscle lengths, increasing filament overlap at maximum muscle extension and allowing for continued force production over greater length changes than in typical striated muscle. Tongue retraction and prey processing, however, are also associated with increased excursions of the hyoid compared with other lizards [17]. Observations of the hyoid retractor muscles (mm. sternohyoideus) contracting more than 50% of their resting length suggest that these muscles may similarly benefit from supercontractile capabilities [17]. Here, we document two chameleon hyoid retractor muscles that are also able to circumvent typical constraints on the ability of skeletal muscle to generate force over extreme length changes, exhibiting similarly broadened length–tension relationships to the m. hyoglossus (figure 2). The m. sternohyoideus superficialis does so by similarly incorporating perforated z-discs (figure 4c,d). Conversely, the m. sternohyoideus profundus is able to do so with solid, uninterrupted z-discs typical of normal striated muscle (figure 4e,f).

Whereas supercontracting muscle occurs widely among invertebrates, including their original description in the barnacle [29,33,34] and a variety of insects [30,35–37,60–63], hitherto the only known vertebrate example of supercontracting muscle is the m. hyoglossus in chameleons [9,31,32]. Our results thereby document the second example of supercontracting muscle among vertebrates, the m. sternohyoideus superficialis of chameleons, which not only exhibits a similar perforated z-disc structure to the m. hyoglossus, but also a similarly broad length–tension relationship. Additionally, these two muscles are aligned in series to each other, which imparts supercontractile characteristics to the entire muscle length between the proximal quarter of the m. accelerator linguae of the tongue where the m. hyoglossus inserts, all the way posteriorly to the ventral side of the xiphisternum, where the m. sternohyoideus superficialis originates [39,41]. Therefore, the extent to which the tongue is able to project out of the mouth in chameleons (up to 250% of body length) is not only mediated by the increased length change that supercontracting muscle properties allow, but also by an increased overall length of the muscle unit, and thus an increased number of sarcomeres in series. Together, these two properties allow for the extreme elongation and projection distances of the chameleon tongue, whereas in the tongue retractors of salamanders, which lack supercontracting muscle but have increased the number of sarcomeres in series, the latter characteristic alone only permits projection distances of up to 80% of their body length [15].

**Figure 6.**
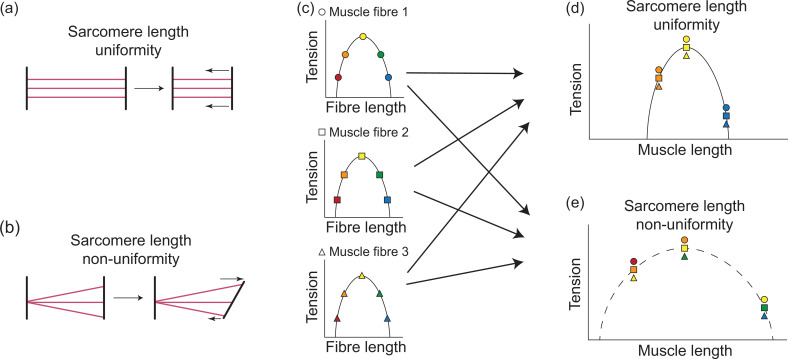
Schematic illustration of sarcomere length non-uniformity or dispersion and its impact on the muscle length–tension relationship. (a) Sarcomere length changes are equal among muscle fibres across the entire muscle when the origin and insertion distances change equally, thus resulting in sarcomere length uniformity. (b) When one muscle attachment site undergoes rotation, as seen in the m. sternohyoideus profundus, some muscle fibres will undergo considerably more lengthening, while others will undergo less lengthening and possibly even shortening, resulting in sarcomere length non-uniformity. (c) Individual muscle fibres (distinguished by different shapes) each have their own length–tension relationships (with different fibre lengths denoted by different colours). (d) Under sarcomere length uniformity, these muscle fibre length–tension relationships will hypothetically produce a similarly shaped length–tension relationship for the entire muscle as all fibres are acting simultaneously on the same portion of their own length–tension relationship (note the different shapes corresponding to each muscle fibre match in colour at any single position along the entire muscle’s length–tension relationship, signifying a similar position on each muscle fibre’s length–tension curve). (e) Under sarcomere length non-uniformity, individual fibres are simultaneously acting on different portions of their length–tension relationships (note shape colours no longer match at any single position along the entire muscle’s length–tension curve, signifying differing positions along each muscle fibre’s length–tension curve), depressing peak contractile force and broadening the muscle length–tension relationship as some sarcomeres are able to move towards and onto the force plateau as others move off and away from it during lengthening and shortening.

That the m. sternohyoideus profundus exhibits a similarly broad length–tension relationship to both the m. hyoglossus and m. sternohyoideus superficialis seems unusual. The m. sternohyoideus profundus lacks the perforated z-discs of supercontracting muscle and should therefore be limited in the extent to which its sarcomeres are able to change length like a typical skeletal muscle. This broadened length–tension relationship is likely due to the broad insertion of this muscle on the posterior distal half of the ceratobranchials [39,41] combined with the significant rotation of the ceratobranchials as the hyoid is protruded from the mouth prior to tongue projection (figure 1a,c). As the tongue is protruded, the hyoid is effectively unfolded and the angle between the ceratobranchials and entoglossal process increases from an acute to obtuse angle [43,44]. With a more localized origin of the m. sternohyoideus profundus anterior to the xiphisternum, this rotation of the ceratobranchials would necessarily cause the sarcomere lengths among muscle fibres inserting down the length of the ceratobranchials to undergo differential shortening and/or elongation relative to their resting position, and cause sarcomere length non-uniformity or dispersion among muscle fibres across the entire muscle (figure 6a,b). Specifically, more ventral fibres of the m. sternohyoideus profundus will undergo considerably more lengthening, while more dorsal fibres will undergo less lengthening, and possibly even shortening, as the hyoid is unfolded and the tongue is protruded from the mouth. In theory, at some orientation between the resting and fully extended position of the hyoid, the muscle fibres of this muscle may exist at lengths that produce consistent sarcomere lengths across the muscle belly, but all other configurations would exhibit non-uniformity to varying degrees. Sarcomere length non-uniformity within myofibrils has been used to explain, among other contractile properties, higher force values than would be expected at long lengths [64]. Sarcomere length non-uniformity, however, is known to occur at all hierarchical levels of muscle [65], and while sarcomere length non-uniformity across the entire muscle belly would depress peak contractile force, it would broaden the length–tension relationship as sarcomeres simultaneously act on different portions of their own length–tension relationship and some sarcomeres are able to move towards and onto the force plateau as others move off and away from it during lengthening and shortening (figure 6c–e). Thus, the m. sternohyoideus profundus in most of the natural orientations of the hyoid is able to functionally maintain force over a wide range of muscle lengths like supercontracting muscle without having anatomically developed the perforated z-discs that are characteristic of supercontracting muscle. Further, this functional convergence with supercontracting muscles through independent mechanisms represents a mechanism by which typical skeletal muscle can achieve supercontractile properties through modifications to fibre orientations and attachments rather than through modification to the sarcomere structure.

Additionally, while the perforated z-discs of supercontracting muscle allowing for actin and myosin filaments from adjacent sarcomeres to interact at short lengths helps explain the force maintenance on the ascending limb of the length–tension relationship, and allows the muscles to rest at short lengths, increasing the distance over which they can lengthen from rest and still maintain filament overlap [31,32], passive tension may limit significant lengthening. Here, we find that both the mm. sternohyoideus superficialis et profundus have significantly longer *L*_20_ values than the m. triceps scapularis and m. hyoglossus and the m. hyoglossus has an average *L*_20_ value slightly longer than the m. triceps scapularis (tables 1 and 2; figure 3a,b). These longer *L*_20_ values indicate that the muscles develop less passive stiffness as they are lengthened, which would decrease the resistance to lengthening as the muscle extends down the descending limb of the length–tension relationship. These shifts in *L*_20_ values are likely due to differences in series and/or parallel elastic elements in these muscles, and may allow for the muscles to operate over a broader range of lengths.

Interestingly, in chameleons the m. hyoglossus is innervated by the hypoglossal nerve (CNXII), while both the mm. sternohyoideus superficialis et profundus are innervated by the first spinal nerve [42]. The hypoglossal nerve is a cranial nerve derived from the occipital somite region [59,66–70] while the spinal nerve derives from the cervical somite area [71]. Previous [9] and present data identify the m. hyoglossus and m. sternohyoideus superficialis as the only two known supercontracting muscles in vertebrates, both found exclusively in the chameleon hyobranchial complex. While the mm. sternohyoideus superficialis et profundus are topographically close and are supplied by spinal nerve 1 [42], early work by Huang *et al.* [66] has shown (using an avian model) that glossal (e.g. m. hyoglossus) and infrahyoid muscles (e.g. m. sternohyoideus superficialis) arise from somites 2−6, while suprahyoid musculature (e.g. m. sternohyoideus profundus) is of cranial paraxial mesoderm origin [72,73]. The presence of supercontraction in the glossal and infrahyoid muscles identified in chameleons (despite being supplied by different nerves) points to the presence of this unique sarcomere ultrastructure potentially due to common embryonic origin from occipital somites through the migration of the hypoglossal cord [66]. Thus, our observation of a deeper origin for the m. sternohyoideus profundus relative to the m. sternohyoideus superficialis (figure 5) agrees with a cranial paraxial mesoderm origin [66,72] and their divergent sarcomere morphology, despite their common name.

These results not only shed light on the mechanistic basis for ballistic tongue projection and extreme elongation of the tongue in chameleons but reveal important developmental and evolutionary patterns associated with supercontracting muscle and extreme muscle elongation. Revealing only the second supercontracting muscle known among vertebrates, the fact that these muscles develop from multiple embryonic sources despite their close anatomical and functional proximity may help inform future studies examining developmental processes associated with muscle and sarcomere specialization. Further, the realization that extreme tongue projection distances in chameleons depend not only on supercontracting muscle properties but also on increases in the number of sarcomeres in series provides important comparative value to other examples of extreme elongation among vertebrates and broader lineages. Finally, the apparent use of sarcomere length non-uniformity to converge on a similar functional mechanism to increase the range of muscle lengths over which a muscle can exert force represents an interesting functional specialization of muscle shape that may be utilized in other systems.

## Data Availability

All muscle contractile data, R Markdown code and source files that support this study are publicly available at the Dryad Digital Repository [74]. Supplementary material is available online [75].
